# An Evaluation of the Immunohistochemical Expression of Mismatch Repair Proteins (MSH2, MSH6, MLH1, and PMS2) in Prostate Adenocarcinoma

**DOI:** 10.7759/cureus.27448

**Published:** 2022-07-29

**Authors:** Saira Javeed, Anila Chughtai, Ghazi Zafar, Fatima Khalid, Ayma Batool, Akhtar S Chughtai

**Affiliations:** 1 Histopathology, Chughtai Institute of Pathology, Lahore, PAK

**Keywords:** anti-programmed cell death protein 1, lynch syndrome, prostate cancer, immunohistochemistry, microsatellite instability, mismatch repair proteins

## Abstract

Background and objective

Mismatch repair (MMR) proteins are an integral part of the cell cycle, and they play an important role in the genomic stability of the microsatellite complex. Microsatellite instability (MSI) is associated with Lynch and multi-tumor syndromes. Identifying patients with Lynch syndrome is essential for screening, early detection, and surveillance of other Lynch syndrome-associated tumors. The role of MMR deficiency is well known in colorectal and endometrial adenocarcinoma. However, the role of MMR deficiency in prostatic adenocarcinoma is a matter of controversy. A few studies have been published to analyze the association between MMR deficiency and prostatic adenocarcinoma. In this study, we used immunohistochemistry to look into the expression of four MMR proteins in prostatic adenocarcinoma: MSH2, MSH6, MLH1, and PMS2.

Methodology

This was a cross-sectional descriptive study involving 74 cases of acinar prostatic adenocarcinoma, diagnosed with hematoxylin & eosin (H&E), over a period of six months between December 2021 and May 2022 at the Chughtai Institute of Pathology in Lahore, Pakistan. We performed the immunohistochemical (IHC) analysis and interpretation of four antibodies, i.e., MSH2, MSH6, MLH1, and PMS2.

Results

In our study, the age of the patients ranged from 50 to 98 years, with a mean age of 67.99 ± 9.59 years. The specimens were collected through transurethral resection of the prostate (TURP), transurethral vaporization of the prostate (TVP), core biopsy, and radical prostatectomy. Isolated loss of each MSH2 and PMS2 was noted in nine cases (12.20%) and MSH6 in two cases (2.70%). There was no loss noted for MLH1. Furthermore, simultaneous loss of MSH2/MSH6 was observed in one case (1.35%).

Conclusion

Our study findings revealed a low frequency of IHC expression of MMR proteins, especially the concurrent loss of paired MMR proteins. However, prostatic adenocarcinoma is associated with the isolated loss of MMR proteins. Thus, the present study does not warrant reflex testing/screening in every case of prostatic adenocarcinoma, because of its low frequency, which is probably suggestive of its sporadic pattern.

## Introduction

Prostate cancer is the second most common malignancy in men worldwide after lung cancer [1]. In Pakistan, since there is no cancer registry at the national level, exact statistics about the incidence and prevalence of prostatic carcinoma are unknown [2]. Furthermore, according to the Punjab Cancer Registry Report in 2018, prostate cancer is the second most common malignancy among men in Pakistan [3]. The incidence and mortality rates of prostate carcinoma strongly correlate with advanced age, and high incidence rates have been reported in men older than 65 years [4]. Initially, the focus of management was on the prognostic factors, including prostate-specific antigen (PSA) levels, Gleason score, grade group, tumor load, and clinical stage. However, with marked advancements in the field of molecular genetics and targeted therapy, these clinicopathological factors are now considered suboptimal from a therapeutic standpoint [4]. Thus, there is an immense need to understand the biological behavior of the disease by recognizing the molecular markers used to predict the aggressive nature of tumors and for the purpose of targeted treatment.

Hereditary non-polyposis colorectal carcinoma (HNPCC), also known as Lynch syndrome, is associated with microsatellite instability (MSI). MSI was detected in the form of loss of mismatch repair (MMR) genes and their proteins. These genes and their proteins are known to cause malignancies, including colorectal, endometrial, gastric, lung, liver, and prostatic carcinoma [5]. MSI is a genetic abnormality, first identified in colorectal carcinoma due to a defect in one or more MMR proteins [6]. Six proteins come under MMR: MSH2, MLH1, PMS2, PMS6, MSH6, and MLH3. These proteins act as sensors to detect DNA damage and repair the defects before the replication of DNA. When MMR proteins are deficient, the process of DNA repair is compromised, leading to abnormal accumulation of damaged DNA, permissive for carcinogenesis [7]. Since MLH1 is paired with PMS2 in terms of its function, and MSH2 is paired with MSH6, their loss is dependent on their paired counterpart. Besides, studies have also reported a loss of MLH1/PMS2 regarding its sporadic pathogenesis in cancers.

In contrast, the loss of MSH2/MSH6, isolated PMS2, and MSH6 favors familial pathways for the pathogenesis of different carcinomas. Defects in MMR proteins are identified by BRAF testing, indirectly through immunohistochemistry, or directly through polymerase chain reaction (PCR), and hypermethylation of MLH1. The absence of BRAF mutations or hypermethylation of MLH1 should prompt an evaluation for Lynch syndrome (next-generation sequencing).

Carcinomas associated with MSI entail a different prognosis; for example, in the case of colorectal or gastric carcinoma, favorable outcomes have been documented, while in the case of non-small cell carcinoma of the lung, poor outcomes have been reported [6]. The role of MSI in prostatic carcinoma in terms of prognosis is relatively new, with limited data available in the literature. Studies have also suggested a link between the loss of MMR proteins and poor prognostic features of prostatic carcinoma [8,9]. In contrast, some contradictory studies have suggested a strong association between the overexpression of MMR development in prostatic cancer and poor outcomes [10,11,12].

The present study aimed to assess the immunohistochemical (IHC) expression of four MMR proteins (MSH2, MSH6, MLH1, and PMS2) in prostatic adenocarcinoma.

## Materials and methods

Ethical statement

The study was conducted after obtaining ethical approval from the Institutional Review Board (IRB) of the Chughtai Institute of Pathology, Lahore, Pakistan (reference letter no: CIP/IRB/1057).

Patient selection

All male patients diagnosed with acinar prostatic adenocarcinoma were included in the study. Patients with prostate ductal adenocarcinoma and prostatic adenocarcinoma with neuroendocrine differentiation were excluded.

Data collection

This was a cross-sectional, descriptive study. Relevant details of 74 cases of acinar prostatic adenocarcinoma diagnosed via hematoxylin & eosin (H&E) over a period of six months between December 2021 to May 2022 were retrieved from the electronic data system (Nexus) of the Chughtai Institute of Pathology. We employed a convenient sampling technique for patient selection. Specimen collection was performed through transurethral resection of the prostate (TURP), transurethral vaporization of the prostate (TVP), core biopsy, and radical prostatectomy. For light microscopy, formalin-fixed paraffin-embedded (FFPE) tissues were stained with H&E. In most cases, the diagnosis was made primarily based on morphology. Yet, in a few difficult cases, immunohistochemistry with p63 and α-methylacyl coenzyme A racemase (AMACR) was applied.

Immunohistochemistry

One representative block with preserved tumor morphology was selected for IHC staining in each case. Staining of all four MMR proteins was performed, MSH2 (Monoclonal Mouse Anti-Human antibody, Clone FE11), MSH6 (Monoclonal Rabbit Anti-Human antibody, Clone EP49), MLH1 (Monoclonal Mouse Anti-Human antibody, Clone ES05), and PMS2 (Monoclonal Rabbit Anti-Human antibody, Clone EP51) (all provided by Agilent Dako, Glostrup, Denmark). After applying the IHC technique, as per standard protocol (Dako Autostainer Link 48, Detection Kit K8002, Agilent Dako), the prepared slides were examined by two consultant pathologists.

Positive external control of normal colonic tissue was run with each batch, and the nuclear positivity of each MMR protein in the benign prostatic epithelium, lymphocytes, stromal, and endothelial cells was considered positive internal control. The nuclear reactivity was classified into two categories: either loss of expression or retained expression. When there was no nuclear reactivity, it was considered a loss of expression (negative in 100% of tumor cells). In comparison, at least 1% nuclear staining of each antibody in tumor cells was considered retained expression (positive in tumor cells) [5]. Those cases where the loss of one or all proteins was observed were categorized as MMR-deficient, while retained MMR proteins were considered MMR-proficient. Patient data, along with biopsy numbers and relevant details, were recorded.

Statistical analysis

The quantitative variable, i.e., age, was analyzed in terms of mean and standard deviation (SD). Qualitative variables such as primary pattern, secondary pattern, Gleason score, grade group, tumor load, perineural invasion (PNI), and loss/retained expression of MMR proteins were calculated as frequencies and percentages.

Data were analyzed and statistical analysis was performed using SPSS Statistics version 22 (IBM Corp., Armonk, NY). Fisher's exact test was employed wherever applicable. A p-value <0.05 was considered statistically significant.

## Results

In total, 74 cases fulfilling the inclusion criteria were enrolled in the present study. Their ages ranged from 50 to 98 years, with a mean age of 67.99 ± 9.59 years. The most frequent type of specimen through which the sample was received was TURP in 62 cases (83.80%), followed by core biopsy in six cases (8.10%), and three cases (4.10%) each for TVP and radical prostatectomy. Among 74 men diagnosed with prostatic adenocarcinoma, 20 (27.0%) had primary Gleason pattern 3, 40 (54.10%) showed Gleason pattern 4, and 14 (18.90%) had Gleason pattern 5 morphology on H&E; the secondary pattern of tumor on H&E with Gleason pattern 3 was observed in 26 (35.10%) cases, followed by Gleason pattern 4 in 31 (41.90%), and Gleason pattern 5 in 17 (23.0%). Regarding grade group, 17 cases (23.0%) belonged to grade group 1, five (6.80%) to grade group 2, nine (12.20%) to grade group 3, 14 (39.20%) to grade group 4, and 29 (39.20%) to grade group 5. PNI was absent in 43 (58.10%) and present in 31 (41.90%) cases. Tumor load ≤50% was seen in 25 (33.80%) cases and that >50% was noted in 49 (66.20%).

**Figure 1:**
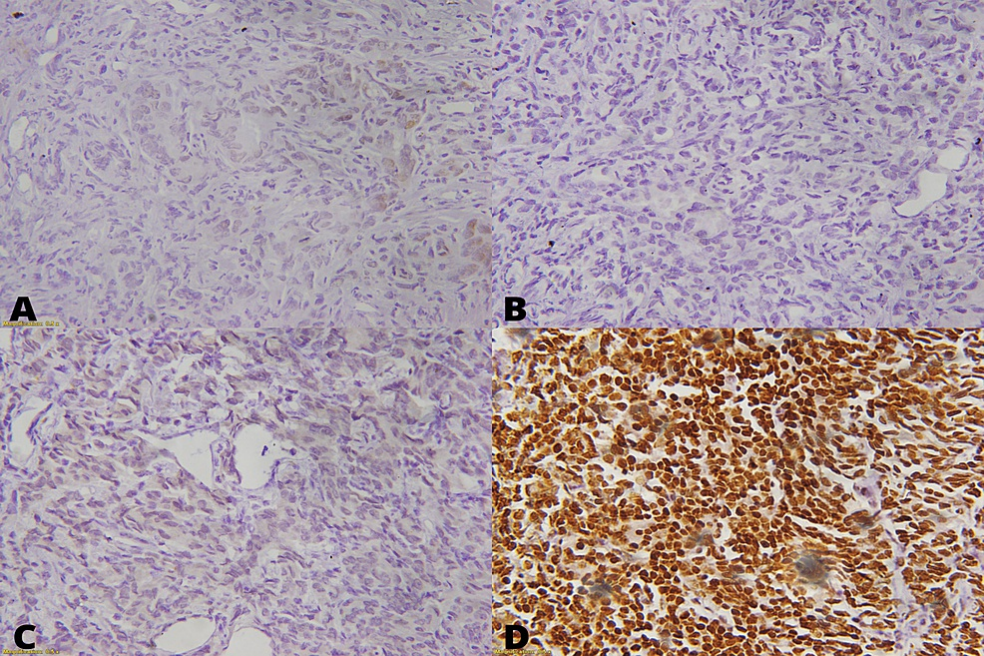
Prostatic adenocarcinoma: (A) nuclear loss of MSH2 40x, (B) nuclear loss of MSH6 40x, (C) nuclear loss of PMS2 40x, and (D) nuclear positivity of MLH1 40x

IHC loss of expression of MSH2, MSH6, MLH1, and PMS2 proteins were seen in nine cases (12.20%), two cases (2.70%), zero case (0.00%), and nine cases (12.20%) respectively. IHC expression and loss are shown in Figure 1.

MMR proteins and their relationship with grade group, PNI, and tumor load are shown in Tables 1, 2, 3 respectively.

**Table 1:** MMR proteins and their relationship with grade group (n=74) MMR: mismatch repair

		Grade group 1, n (%)	Grade group 2, n (%)	Grade group 3, n (%)	Grade group 4, n (%)	Grade group 5, n (%)	P-value
MSH2	Loss of expression	1 (1.35%)	0	2 (2.70%)	0	6 (8.11%)	0.21
	Retained expression	16 (21.62%)	5 (6.76%)	7 (9.46%)	14 (18.92%)	23 (31.08%)	
MSH6	Loss of expression	1 (1.35%)	0	0	0	1 (1.35%)	1.0
	Retained expression	16 (21.62%)	5 (6.76%)	9 (12.16%)	14 (18.92%)	28 (37.84%)	
MLH1	Loss of expression	0	0	0	0	0	
	Retained expression	17 (22.98%)	5 (6.76%)	9 (12.16%)	14 (18.92%)	29 (39.19%)	
PMS2	Loss of expression	2 (2.70%)	0	1 (1.35%)	1 (1.35%)	5 (6.76%)	0.97
	Retained expression	15 (20.27%)	5 (6.76%)	8 (10.81%)	13 (17.57%)	24 (32.43%)	

**Table 2:** MMR proteins and their relationship with perineural invasion (n=74) MMR: mismatch repair

		Perineural invasion absent, n (%)	Perineural invasion present, n (%)	P-value
MSH2	Loss of expression	2 (2.70%)	7 (9.46%)	0.02
	Retained expression	41 (55.41%)	24 (32.43%)	
MSH6	Loss of expression	1 (1.35%)	1 (1.35%)	0.66
	Retained expression	42 (56.76%)	30 (40.54%)	
MLH1	Loss of expression	0	0	
	Retained expression	43 (58.10%)	31 (41.90%)	
PMS2	Loss of expression	7 (9.46%)	2 (2.70%)	0.18
	Retained expression	36 (48.65%)	29 (39.19%)	

**Table 3:** MMR proteins and their relationship with tumor load (n=74) MMR: mismatch repair

		Tumor load ≤50%, n (%)	Tumor load >50%, n (%)	P-value
MSH2	Loss of expression	1 (1.35%)	8 (10.81%)	0.12
	Retained expression	24 (32.44%)	41 (55.40%)	
MSH6	Loss of expression	2 (2.70%)	0 (0%)	0.10
	Retained expression	23 (31.08%)	49 (66.22%)	
MLH1	Loss of expression	0	0	
	Retained expression	25 (33.80%)	49 (66.20%)	
PMS2	Loss of expression	3 (4.05%)	6 (8.11%)	0.64
	Retained expression	22 (29.73%)	43 (58.11%)	

The frequency of loss of MMR proteins in relation to age, Gleason score, grade group, PNI, and tumor load is compared in Table 4.

**Table 4:** Frequency of loss of MMR proteins in relation to age, Gleason score, grade group, perineural invasion, and tumor load (n=74) MMR: mismatch repair

		MMR-deficient	MMR-proficient		P-value
Age	≤60 years	5	11		0.28
	>60 years	12	46		
Gleason score	6	3	14		0.21
	7	3	9		
	8	1	15		
	9	9	18		
	10	1	1		
Grade group	1	3	14		0.19
	2	0	5		
	3	3	6		
	4	1	13		
	5	10	19		
Perineural invasion	Absent	9	34		0.41
	Present	8	23		
Tumor load	≤50%	4	21		0.24
	>50%	13	36		

## Discussion

In recent years, men with prostatic carcinoma associated with Lynch syndrome and sporadic cases have been linked with defects in MMR proteins. Worldwide, the detection of MMR proteins by IHC is routinely done in colorectal and endometrial adenocarcinoma and even in prostatic adenocarcinoma. Nonetheless, in Pakistan, there is no routine evaluation for MMR proteins detected in prostatic adenocarcinoma or even in recognized MMR-deficient tumors, i.e., colorectal and endometrial adenocarcinoma. In one study by Hashmi et al. and another by Qasim et al., a high frequency of MMR deficiency in colorectal and endometrial adenocarcinoma was observed, respectively [13,14]. To the best of our knowledge, no study conducted in Pakistan has analyzed the link between MMR protein deficiencies and prostatic carcinoma.

In the current study, we evaluated the IHC expression of MSH2, MSH6, MLH1, and PMS2 proteins in terms of their deficiency, single or combined, in prostatic adenocarcinoma. We also analyzed their relationship with respect to age, Gleason score, grade group, PNI, and tumor load.

The present study revealed an association between the incidence of prostatic adenocarcinoma and advanced age (mean age of 68 years), which is in line with the findings of Wilczak et al. [4]. The majority of cases in which MMR deficiency was seen had a Gleason score of 9-10 and belonged to grade group 5 (13.51%). These results described the association of loss of MMR proteins with the high-grade group, although statistically insignificant, which aligns with the study by Guedes et al., in which they found the maximum loss of MSH2 protein in prostatic adenocarcinoma with high Gleason score/grade group [15]. In contrast, Albero-González et al. found no similar association. As a matter of fact, very few studies have correlated the overexpression of MMR proteins with aggressive behavior in terms of higher clinical stage, score, and grade [4].

In our study, no significant relationship between the loss of MMR proteins and the absence or presence of PNI was noted. Cases with >50% tumor load demonstrated a high frequency of MMR deficiency (17.56%) in comparison to cases with ≤50% tumor load (5.40%). In the meantime, most of the studies have not correlated MMR deficiency with PNI and tumor load. Additionally, our study found a low frequency of the loss of MSH2, MSH6, and PMS2, i.e., 12.20%, 2.70%, and 12.20%, respectively, which is statistically insignificant. There was no loss for MLH1. These low frequencies match with the results of the studies conducted in the past [4,7,9], where deficiency of MMR proteins was noted in a few cases. These isolated losses of MMR proteins are linked to sporadic cases. However, a concurrent loss of MSH2/MSH6 was seen in only one case in our study, which reflects an association with Lynch syndrome. Knowing the pathogenesis is of great significance in predicting the prognosis. Studies have revealed a poor prognosis for sporadic prostatic adenocarcinoma [16]. However, in a study by Schweizer et al., cases of prostatic adenocarcinoma with MMR deficiency resulted in favorable outcomes. It is because MMR-deficient patients have shown more sensitivity to anti-programmed cell death protein 1 (anti-PD-1) and its ligand programmed death-ligand 1 (PD-L1) therapy, suggesting predictive behavior for treatment response [17].

In the case of colorectal carcinoma and other malignancies associated with MSI, the role of anti-PD-1 and PD-L1 for treatment purposes is well-known and accepted by the United States Food and Drug Administration. On the contrary, in the case of prostate cancer, the role of anti-PD1 and PD-L1 has yet to be established, primarily due to a scarcity of data [18].

Limitations

There are a few limitations to the present study. Data were collected retrospectively, and preoperative serum PSA levels were unavailable. We could not see a correlation between serum PSA levels and the loss of MMR proteins. Besides, employing IHC alone to assess the loss of MMR proteins and label its association with Lynch syndrome is not a standard protocol.

## Conclusions

Our study revealed a low frequency of IHC expression of MMR proteins, especially the concurrent loss of paired MMR proteins. However, prostatic adenocarcinoma is associated with isolated loss of MMR proteins. Thus, the findings of the present study do not warrant reflex testing/screening in every case of prostatic adenocarcinoma, because of its low frequency, which is probably suggestive of its sporadic pattern.
